# Uptake of the first to fifth doses of coronavirus disease 2019 vaccine in individuals with chronic lymphocytic leukaemia: A nationwide cohort study in Sweden

**DOI:** 10.1002/jha2.1077

**Published:** 2025-01-06

**Authors:** Pontus Hedberg, Lisa Blixt, Fredrik Granath, Peter Bergman, Christina Carlander, Soo Aleman, Lotta Hansson, the CLHIP study group

**Affiliations:** ^1^ Department of Medicine Huddinge Karolinska Institutet Stockholm Sweden; ^2^ Department of Oncology‐Pathology Karolinska Institutet Stockholm Sweden; ^3^ Department of Haematology Karolinska University Hospital Stockholm Sweden; ^4^ Department of Medicine Solna Clinical Epidemiology Division Karolinska Institutet Stockholm Sweden; ^5^ Department of Laboratory Medicine Karolinska Institutet Stockholm Sweden; ^6^ Department of Clinical Immunology and Transfusion Medicine Karolinska University Hospital Stockholm Sweden; ^7^ Department of Infectious Diseases Karolinska University Hospital Stockholm Sweden; ^8^ Department of Medical Epidemiology and Biostatistics Karolinska Institutet Stockholm Sweden

**Keywords:** CLL, COVID‐19, vaccination uptake

## Abstract

**Objectives:**

Patients with chronic lymphocytic leukaemia (CLL) have an increased risk of severe coronavirus disease 2019 (COVID‐19) as well as impaired responses to COVID‐19 vaccination, which may be overcome by repeated booster vaccinations. Our objective was to explore the uptake of the COVID‐19 vaccine in this population since records of this are scarce.

**Methods:**

In this nationwide cohort study, we used multiple population‐based health and sociodemographic registries to study COVID‐19 vaccine uptake in individuals with CLL in Sweden, from 27 December 2020 to 28 February 2023.

**Results:**

A total of 6304 individuals were included. The cumulative incidence (95% confidence interval) at the end of the study period was 95%, 94%, 88%, 78% and 56% for the first, second, third, fourth and fifth doses, respectively. The uptake was significantly higher compared with the age‐standardized nationwide uptake. However, there were large disparities, especially for the fourth and fifth doses, across different age groups, birth regions, and income quartiles. These differences were especially pronounced in intersectional analyses, where individuals born abroad in the lowest income quartile had a vaccine uptake of only 49% and 24% for the fourth and fifth doses, respectively.

**Conclusions:**

Even though uptake was generally high in individuals with CLL, it seems to be declining from dose three and onwards, and there are significant sociodemographic disparities in vaccine uptake.

## 1 INTRODUCTION

Chronic lymphocytic leukaemia (CLL) is a haematological malignancy estimated to affect around 0.06% of individuals in Europe and the United States [1]. Both disease‐related and treatment‐related immune defects observed in individuals with CLL lead to an increased risk of severe coronavirus disease 2019 (COVID‐19) [2, 3, 4, 5, 6]. In addition, individuals with CLL have an impaired response to COVID‐19 vaccinations and multiple vaccine doses are often required to increase seroconversion rates and anti‐spike antibody levels [2, 7–9]. This has been demonstrated in recent studies, including a Swedish cohort study and a large multicentre study coordinated by the European Research Initiative on CLL (ERIC) [10, 11]. Previous studies have observed sociodemographic disparities in COVID‐19 vaccine uptake across different populations, however, studies focused on CLL patients’ vaccine uptake are scarce [12, 13, 14, 15, 16, 17, 18]. A cohort study from England, including 97,707 individuals with a blood cancer diagnosis, found area‐level social and individual‐level ethnic disparities in COVID‐19 vaccine uptake and a trend for lower uptake with each subsequent vaccine dose [18]. However, that study was mainly focused on blood cancer as a combined category and did not analyse individual‐level social disparities for specific blood cancer types. To the best of our knowledge, no previous study has focused specifically on understanding the magnitude of individual‐level sociodemographic disparities in COVID‐19 vaccine uptake in patients with CLL. Such characterizations are crucial to guide strategies for future vaccination campaigns and epidemic scenarios. Accordingly, the aim of this nationwide study was to investigate how sociodemographic factors, comorbidities, and CLL status possibly influenced the uptake of the first to fifth doses of the COVID‐19 vaccine in adults diagnosed with CLL in Sweden.

## 2 METHODS

### 2.1 Study design and data sources

We conducted a nationwide register‐based cohort study of COVID‐19 vaccine uptake in adults aged 18–90 years diagnosed with CLL in Sweden. The study was approved by the Swedish Ethical Review Authority (IDs 2022‐01793‐01 and 2023‐05877‐02). Data from six national registers and other sources with high coverage were used: i) National Quality Registry for CLL, ii) Total Population Register (TPR), iii) National Vaccination Register (NVR), iv) Swedish Cause of Death Register (SCDR), v) Longitudinal Integrated Database for Health Insurance and Labour Market Studies (LISA) and vi) Swedish Prescribed Drug Register (SPDR). Individuals diagnosed with CLL in Sweden are reported to the National Quality Registry for CLL (reporting coverage of 97%), which is part of the Information Network for Cancer Care (INCA) platform, maintained by the Regional Cancer Centres (RCC) in Sweden with the aim of improving cancer care throughout the nation. TPR is maintained by Statistics Sweden and contains data on birth, death, emigration and immigration, sex, and country of birth for the Swedish population [19]. All vaccinations within national vaccination programs and vaccinations against COVID‐19 should according to Swedish law be reported to the NVR, which is governed by the Public Health Agency of Sweden (PHAS) [20]. SCDR is an almost complete register of all deaths in Sweden, maintained by the National Board of Health and Welfare [21]. LISA is maintained by Statistics Sweden and contains data on around 500 variables on individuals including civil status, educational status, income and allowances [22]. SPDR is maintained by the National Board of Health and Welfare and contains data for all dispensed prescriptions in Sweden [23].

### 2.2 Study population

All individuals born from 1930 to 2003 diagnosed with CLL any time up until the end of the study, 28 February 2023, who were alive and living in Sweden any time from 1 February 2020 to the end of the study were considered for inclusion. Among these individuals, we included those alive and living in Sweden on 27 December 2020, the date of the first COVID‐19 vaccination in Sweden. Furthermore, we only included individuals who were diagnosed with CLL up until 30 November 2022 to allow for 90 days of follow‐up from the date of the CLL diagnosis to the end of the study. A distinction was made between individuals diagnosed with CLL before and after the date of the first COVID‐19 vaccination in Sweden. This was done since individuals diagnosed with CLL before the date of the first COVID‐19 vaccination could consider their underlying CLL in all decisions to get vaccinated, whereas individuals diagnosed after per definition were not aware of their disease during the entire vaccination programme and thus could have made different choices.

### 2.3 The Swedish COVID‐19 vaccination program

The Swedish COVID‐19 vaccination program was designed by PHAS, commissioned by the Swedish government, and included a priority system for initial vaccination. The aim was to prioritize those with the highest risk of severe disease and death from COVID‐19. Old age was regarded as the most significant risk factor for severe COVID‐19, based on available Swedish and international data, and therefore an “oldest first” policy ruled throughout the program, followed by certain comorbidities [24, 25, 26, 27].

The vaccination program was funded by the Swedish government; hence, vaccination has been free of charge for all Swedish citizens as well as for any institutions performing vaccinations. Vaccination was readily available for all citizens successively according to PHAS's priority guidelines, and individuals were informed when vaccination was available to them.

During 2020–2022 Sweden used the vaccines provided by Pfizer BioNTech (63%), Moderna (29%), AstraZeneca (4%) and Novavax (4%) [28]. At the very beginning of the vaccination program, there were some limitations to the extent of vaccination due to the inadequate supply of vaccines.

National Guidelines on CLL were updated with specific recommendations stating that all CLL patients should be encouraged to vaccinate against SARS‐CoV‐2 as soon as possible after CLL diagnosis. Furthermore, close relatives should be vaccinated since patients with CLL tend to have an impaired effect of conventional vaccines [29].

After the primary vaccination phase (two doses), additional booster vaccination has been recommended for people with various immune defects continuously from 8 weeks after primary vaccination, with doses three to five available gradually from 1 September 2021, as described below. However, booster vaccination has not been as closely monitored as the primary vaccination phase but rather left to individual responsibility, and guidelines on booster vaccination have been updated continuously.

### 2.4 Outcomes

The uptake of the first to fifth doses of COVID‐19 vaccines was analysed. Follow‐up started on 27 December 2020 and finished by date of death, date of moving out from Sweden, or 28 February 2023, whichever occurred first. For individuals who were diagnosed with CLL after 27 December 2020, follow‐up started at the date of diagnosis. For the second dose, at least 19 days should have passed since the first dose for BNT162b2 and NVX‐CoV2372, and 25 days for mRNA‐1273, AZD1222 and Ad26.COV2.S as previously described [30]. Third doses were considered from 1 September 2021 as described in publicly available analyses of COVID‐19 vaccinations from PHAS [31]. For the fourth and fifth doses, these dates were from 21 January 2022 and 15 August 2022, respectively. For the third to fifth doses, at least 8 weeks (56 days) should have passed since the preceding dose as described in the analyses from PHAS.

### 2.5 Independent variables

Data on age, sex, region of birth, age‐standardized income quartile, number of prescribed drug types, and CLL status were collected. Age was categorized as 18–64, 65–79 and 80–90 years. Region of birth was categorized as being born in Sweden or being born outside of Sweden. Income quartiles for each birth year in the entire Swedish population were first calculated, and the study participants were then classified into income quartiles based on their income in relation to these population‐based quartiles. The income variable was the total sum of incomes, including earned income, age‐related pensions, compensation from unemployment insurance, sickness allowance, etc. The number of prescribed drug types were the number of the following drug types prescribed from 27 December 2019 to 27 December 2020 (the last year before the start of the study): Antidiabetic drugs, antihypertensive drugs, antithrombotic agents, drugs for obstructive airway disease, heart disease drugs, immunosuppressive drugs, and lipid modifying agents. CLL status included both time since diagnosis (>5 years since diagnosis and ≤5 years since diagnosis) and treatment status (Treated or Untreated). For CLL treatment status, the following drug classes were considered: Anti‐CD20 antibodies, BTK inhibitors, BCL‐2 inhibitors, chemotherapy, corticosteroids and PI3K inhibitors. For BTK inhibitors, BCL‐2 inhibitors, chemotherapy, and PI3K inhibitors, all treatments given before 27 December 2020 (start of study) were considered. For anti‐CD20 antibodies, treatments given from 27 December 2019 to 27 December 2020 (the last year before the start of the study) were considered. For corticosteroids, treatments given from 28 September 2020 to 27 December 2020 (last 3 months) were considered. Corticosteroid injections and prescriptions with a daily dose corresponding to less than 10 mg of prednisone were not considered. Individuals who had not received any of these treatments were classified as untreated. All study variables are described in more detail in Table S1.

### 2.6 Statistical methods

First, we described the baseline characteristics of the study population. Individuals diagnosed with CLL before and after the date of the first COVID‐19 vaccination in Sweden (27 December 2020) were also described separately. Continuous variables were presented as median (interquartile range [IQR]) and categorical variables were reported as frequencies (percentages).

We then analysed the COVID‐19 vaccine uptake among individuals diagnosed with CLL before 27 December 2020. The cumulative incidences of uptake of the first to fifth doses were analysed using the Aalen and Johansen estimator, with death and emigration before each vaccine dose as competing events [32]. To understand if the vaccine uptake in individuals with CLL was higher than in the general population, the nationwide uptake was then standardized to the age distribution in the CLL study population. This was assessed by calculating the vaccine uptake in age groups < 40, 40–49, 50–59, 60–69, 70–79 and 80–90 years using data from NVR, with population denominators retrieved from Statistics Sweden. The vaccine uptake in each age group was then multiplied by the proportion of CLL patients in the study population belonging to each age group and finally added together to obtain the age‐standardized vaccine uptake. This calculation was done separately for the first to fifth doses.

The cumulative incidences (with 95% confidence intervals [CI]) of receipt of the first to fifth doses at the end of follow‐up were then analysed for each of the independent variables listed in the ‘Independent variables’ section. Cumulative incidence curves, along with the corresponding *P* values obtained from Gray's test, were also presented. Statistical significance was assessed at the 0.05 alpha level. To further understand the combined effects of the independent variables, which seemed to have the largest impact on vaccine uptake (age group, region of birth, and income quartile), two intersectional analyses were conducted. The first analysis combined region of birth (Sweden, Outside Sweden) and income quartile (Quartile 1 [lowest income], Quartile 2‐Quartile 4), resulting in four intersectional strata. The cumulative incidences (with 95% CI) of receipt of the first to fifth doses at the end of follow‐up were analysed for the four strata. The second analysis also included age groups (18 to 64 years, 65 to 79 years and 80–90 years), thus including 12 intersectional strata. Cumulative incidence curves, along with the corresponding *p*‐values obtained from Gray's test, were also presented for both analyses.

The vaccine uptake at the end of the study in individuals diagnosed with CLL during the study period was then compared with individuals diagnosed with CLL before the study period.

The data sources contained very low levels of missing data for the region of birth (*n* = 1) and age‐standardized income quartile (*n* = 1). These two individuals were excluded from analyses related to these two variables. All other variables had no missing data. Analyses were conducted using R version 4.1.0.

## 3 RESULTS

### 3.1 Study population

**FIGURE 1:**
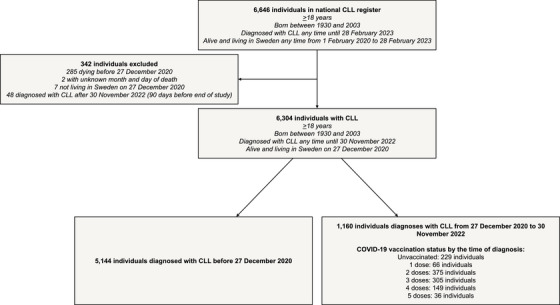
Study flow chart. CLL, Chronic lymphocytic leukaemia; COVID‐19, Coronavirus disease 2019.

Of the 6646 individuals with CLL considered for inclusion, 6304 were finally included (Figure 1). A total of 5144 individuals had received a CLL diagnosis before 27 December 2020. Among the 1160 individuals receiving a CLL diagnosis after 27 December 2020, 20% (*n* = 229) were unvaccinated and 42% (*n* = 490) had received three doses or more at the time of diagnosis.

### 3.2 Baseline characteristics

The median (IQR) age was 74 (67–80) years and 62% (*n* = 3876) were males (Table 1). A total of 12% (*n* = 779) were born outside of Sweden and 30% (*n* = 1859) were in the highest age‐standardized income quartile. Among the 5144 individuals diagnosed with CLL before the study period, 49% (*n* = 2502) had received a diagnosis >5 years ago and 51% (*n* = 2642) had received a diagnosis ≤5 years ago. The proportion considered treated (as described in the Methods section) at the study start was 60% (*n* = 1509) for those having received a diagnosis >5 years ago and 80% (*n* = 2103) for those having received a diagnosis ≤5 years ago. Overall, the baseline characteristics were similar for individuals who received the CLL diagnosis before compared with during the study period.

**TABLE 1:** Baseline characteristics of the study population.

	Overall (*n* = 6304)	Diagnosed with CLL before the start of the study (*n* = 5144)	Diagnosed with CLL during the study period (*n* = 1160)
Age, years (median [IQR])	74.0 [67.0, 80.0]	74.0 [68.0, 80.0]	72.0 [64.0, 78.0]
18–64 years	1226 (19.4)	919 (17.9)	307 (26.5)
65–79 years	3499 (55.5)	2877 (55.9)	622 (53.6)
80–90 years	1579 (25.0)	1348 (26.2)	231 (19.9)
Male sex	3876 (61.5)	3134 (60.9)	742 (64.0)
Region of birth			
Asia and Pacific	17 (0.3)	12 (0.2)	5 (0.4)
Eastern Europe and Central Asia	217 (3.4)	169 (3.3)	48 (4.1)
Latin America and the Caribbean	8 (0.1)	6 (0.1)	2 (0.2)
Middle East and North Africa	155 (2.5)	114 (2.2)	41 (3.5)
North America	12 (0.2)	9 (0.2)	3 (0.3)
Sub‐Saharan Africa	11 (0.2)	9 (0.2)	2 (0.2)
Sweden	5524 (87.6)	4535 (88.2)	989 (85.3)
Western Europe except Sweden	359 (5.7)	290 (5.6)	69 (5.9)
Missing	1 (0.0)	0 (0.0)	1 (0.1)
Age‐standardized income quartile			
1 (Lowest income)	1314 (20.8)	1060 (20.6)	254 (21.9)
2	1446 (22.9)	1186 (23.1)	260 (22.4)
3	1684 (26.7)	1397 (27.2)	287 (24.7)
4 (Highest income)	1859 (29.5)	1500 (29.2)	359 (30.9)
Missing	1 (0.0)	0 (0.0)	1 (0.0)
Prescription drugs (not CLL‐specific)			
Antidiabetics	991 (15.7)	827 (16.1)	164 (14.1)
Antihypertensives	3873 (61.4)	3203 (62.3)	670 (57.8)
Antithrombotics	2408 (38.2)	2044 (39.7)	364 (31.4)
Heart disease drugs	537 (8.5)	447 (8.7)	90 (7.8)
Immunosuppressive drugs	785 (12.5)	753 (14.6)	32 (2.8)
Lipid‐modifiers	2162 (34.3)	1773 (34.5)	389 (33.5)
Obstructive airway disease drugs	892 (14.1)	735 (14.3)	157 (13.5)
Number of prescribed drug types			
0	1476 (23.4)	1116 (21.7)	360 (31.0)
1	1463 (23.2)	1204 (23.4)	259 (22.3)
2	1239 (19.7)	1046 (20.3)	193 (16.6)
3 or more	2126 (33.7)	1778 (34.6)	348 (30.0)
CLL status on 27 December 2020 ^a^			
>5 years since diagnosis, Untreated	NA	993 (19.3)	NA
>5 years since diagnosis, Treated	NA	1509 (29.3)	NA
≤5 years since diagnosis, Untreated	NA	539 (10.5)	NA
≤5 years since diagnosis, Treated	NA	2103 (40.9)	NA
COVID‐19 vaccination status at end of follow‐up			
Unvaccinated	288 (4.6)	253 (4.9)	35 (3.0)
One dose	52 (0.8)	44 (0.9)	8 (0.7)
Two doses	373 (5.9)	313 (6.1)	60 (5.2)
Three doses	697 (11.1)	545 (10.6)	152 (13.1)
Four doses	1383 (21.9)	1090 (21.2)	293 (25.3)
Five doses	3511 (55.7)	2899 (56.4)	612 (52.8)

Abbreviations: CLL, Chronic lymphocytic leukaemia; COVID‐19, Coronavirus disease 2019; IQR, Interquartile range; NA, Not applicable.

^a^ See the Methods section and Table S1 for more details on definitions used for treatment.

### 3.3 Characteristics of individuals diagnosed with CLL before 27 December 2020 by COVID‐19 vaccine uptake

The age and sex distributions were similar across the vaccine uptake at the end of follow‐up (Table 2). Despite being born in Sweden being more uncommon among unvaccinated individuals compared to those having received one dose or more, around 75% of unvaccinated individuals were born in Sweden. The proportion dying during follow‐up was 43% (109/253) for unvaccinated individuals, 68% (30/44) for individuals having received one dose, 58% (181/313) for individuals having received two doses, 31% (166/545) for individuals having received three doses, 14% (148/1090) for individuals having received four doses, and 2% (65/2899) for individuals having received five doses. The percentage having a COVID‐19 diagnosis code registered according to the death certificate was above 10% for individuals who were unvaccinated or had received one dose compared with less than 5% for individuals having received two doses or more.

**TABLE 2:** Baseline characteristics of individuals diagnoses with chronic lymphocytic leukaemia (CLL) before 27 December 2020 by coronavirus disease 2019 (COVID‐19) vaccine uptake at the end of follow‐up.

	Overall (*n* = 5144)	Unvaccinated (*n* = 253)	One dose (*n* = 44)	Two doses (*n* = 313)	Three doses (*n* = 545)	Four doses (*n* = 1090)	Five doses (*n* = 2899)
Age, years (median [IQR])	74.0 [68.0, 80.0]	73.0 [64.0, 81.0]	75.5 [71.0, 83.0]	76.0 [67.0, 83.0]	73.0 [63.0, 80.0]	73.0 [64.0, 79.0]	75.0 [69.0, 80.0]
18–64 years	919 (17.9)	65 (25.7)	7 (15.9)	69 (22.0)	149 (27.3)	284 (26.1)	345 (11.9)
65–79 years	2877 (55.9)	113 (44.7)	21 (47.7)	130 (41.5)	253 (46.4)	564 (51.7)	1796 (62.0)
80–90 years	1348 (26.2)	75 (29.6)	16 (36.4)	114 (36.4)	143 (26.2)	242 (22.2)	758 (26.1)
Male sex	3134 (60.9)	152 (60.1)	26 (59.1)	189 (60.4)	341 (62.6)	658 (60.4)	1768 (61.0)
Region of birth							
Asia and Pacific	12 (0.2)	1 (0.4)	0 (0.0)	2 (0.6)	2 (0.4)	4 (0.4)	3 (0.1)
Eastern Europe and Central Asia	169 (3.3)	33 (13.0)	0 (0.0)	21 (6.7)	25 (4.6)	37 (3.4)	53 (1.8)
Latin America and the Caribbean	6 (0.1)	2 (0.8)	0 (0.0)	0 (0.0)	0 (0.0)	1 (0.1)	3 (0.1)
Middle East and North Africa	114 (2.2)	11 (4.3)	4 (9.1)	21 (6.7)	29 (5.3)	29 (2.7)	20 (0.7)
North America	9 (0.2)	0 (0.0)	1 (2.3)	0 (0.0)	0 (0.0)	2 (0.2)	6 (0.2)
Sub‐Saharan Africa	9 (0.2)	2 (0.8)	0 (0.0)	1 (0.3)	3 (0.6)	2 (0.2)	1 (0.0)
Sweden	4535 (88.2)	187 (73.9)	38 (86.4)	247 (78.9)	457 (83.9)	947 (86.9)	2659 (91.7)
Western Europe except Sweden	290 (5.6)	17 (6.7)	1 (2.3)	21 (6.7)	29 (5.3)	68 (6.2)	154 (5.3)
Age‐standardized income quartile							
1 (Lowest income)	1060 (20.6)	97 (38.3)	12 (27.3)	95 (30.4)	144 (26.4)	234 (21.5)	478 (16.5)
2	1186 (23.1)	56 (22.1)	14 (31.8)	64 (20.4)	142 (26.1)	269 (24.7)	641 (22.1)
3	1397 (27.2)	59 (23.3)	9 (20.5)	85 (27.2)	156 (28.6)	294 (27.0)	794 (27.4)
4 (Highest income)	1500 (29.2)	40 (15.8)	9 (20.5)	69 (22.0)	103 (18.9)	293 (26.9)	986 (34.0)
Missing	1 (0.0)	1 (0.4)	0 (0.0)	0 (0.0)	0 (0.0)	0 (0.0)	0 (0.0)
Prescription drugs (not CLL‐specific)							
Antidiabetics	827 (16.1)	41 (16.2)	5 (11.4)	64 (20.4)	85 (15.6)	196 (18.0)	436 (15.0)
Antihypertensives	3203 (62.3)	142 (56.1)	32 (72.7)	199 (63.6)	343 (62.9)	666 (61.1)	1821 (62.8)
Antithrombotics	2044 (39.7)	106 (41.9)	18 (40.9)	146 (46.6)	219 (40.2)	379 (34.8)	1176 (40.6)
Heart disease drugs	447 (8.7)	21 (8.3)	4 (9.1)	43 (13.7)	47 (8.6)	77 (7.1)	255 (8.8)
Immunosuppressive drugs	753 (14.6)	48 (19.0)	8 (18.2)	50 (16.0)	78 (14.3)	177 (16.2)	392 (13.5)
Lipid‐modifiers	1773 (34.5)	67 (26.5)	12 (27.3)	105 (33.5)	187 (34.3)	359 (32.9)	1043 (36.0)
Obstructive airway disease drugs	735 (14.3)	40 (15.8)	9 (20.5)	59 (18.8)	83 (15.2)	173 (15.9)	371 (12.8)
Number of prescribed drug types							
0	1116 (21.7)	69 (27.3)	6 (13.6)	60 (19.2)	115 (21.1)	252 (23.1)	614 (21.2)
1	1204 (23.4)	54 (21.3)	11 (25.0)	67 (21.4)	123 (22.6)	255 (23.4)	694 (23.9)
2	1046 (20.3)	41 (16.2)	10 (22.7)	57 (18.2)	121 (22.2)	218 (20.0)	599 (20.7)
3 or more	1778 (34.6)	89 (35.2)	17 (38.6)	129 (41.2)	186 (34.1)	365 (33.5)	992 (34.2)
CLL status on 27 December 2020^a^							
>5 years since diagnosis, Untreated	993 (19.3)	59 (23.3)	8 (18.2)	55 (17.6)	103 (18.9)	217 (19.9)	551 (19.0)
>5 years since diagnosis, Treated	1509 (29.3)	76 (30.0)	12 (27.3)	83 (26.5)	158 (29.0)	313 (28.7)	867 (29.9)
≤5 years since diagnosis, Untreated	539 (10.5)	25 (9.9)	3 (6.8)	46 (14.7)	66 (12.1)	108 (9.9)	291 (10.0)
≤5 years since diagnosis, Treated	2103 (40.9)	93 (36.8)	21 (47.7)	129 (41.2)	218 (40.0)	452 (41.5)	1190 (41.0)
Censoring reason							
Dying	699 (13.6)	109 (43.1)	30 (68.2)	181 (57.8)	166 (30.5)	148 (13.6)	65 (2.2)
Moving out of Sweden	2 (0.0)	0 (0.0)	0 (0.0)	2 (0.6)	0 (0.0)	0 (0.0)	0 (0.0)
Administrative censoring^b^	4443 (86.4)	144 (56.9)	14 (31.8)	130 (41.5)	379 (69.5)	942 (86.4)	2834 (97.8)
COVID‐19 death^c^	107 (2.1)	28 (11.1)	6 (13.6)	14 (4.5)	25 (4.6)	21 (1.9)	13 (0.4)
Cancer death^d^	540 (10.5)	79 (31.2)	26 (59.1)	141 (45.0)	131 (24.0)	120 (11.0)	43 (1.5)

Abbreviations: CLL, Chronic lymphocytic leukaemia; COVID‐19, Coronavirus disease 2019; IQR, Interquartile range.

^a^ See the Methods section and Table S1 for more details on definitions used for treatment.

^b^ Administrative censoring was occurring on 28 February 2023.

^c^ Having a U07.1 or U07.2 diagnosis code registered according to the death certificate.

^d^ Having a cancer diagnosis code registered according to the death certificate.

### 3.4 COVID‐19 vaccine uptake in individuals diagnosed with CLL before 27 December 2020

**FIGURE 2:**
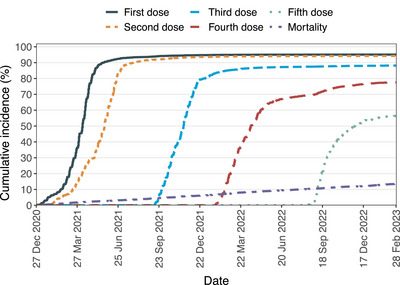
Cumulative incidences of uptake of first to fifth dose and all‐cause mortality. **Note**: Mortality and moving out of Sweden were considered competing events for each specific vaccine dose, that is, mortality before or after having received a first dose but not having received a second dose was a competing event for the analysis of the second dose, but not for the first dose. For the all‐cause mortality, moving out of Sweden was considered a competing event.

The cumulative incidence (95% CI) at the end of the study period was 95% (94–96) for the first dose, 94% (94–95) for the second dose, 88% (87–89) for the third dose, 78% (76–79) for the fourth dose, and 56% (55‐58) for the fifth dose (Figure 2). These cumulative incidences and 95% CIs were all higher than the age‐standardized nationwide uptake (92% for the first dose, 91% for the second dose, 85% for the third dose, 70% for the fourth dose and 52% for the fifth dose). A total of 14% (*n* = 699) died during the study period, of which 91% (*n* = 634) died before having received a fifth dose. Of the 699 individuals who died during the study period, 15% (*n* = 107) had a COVID‐19 diagnosis registered and 77% (*n* = 540) had a cancer diagnosis registered, according to the death certificate. Only two individuals moved out of Sweden during the study period.

**FIGURE 3:**
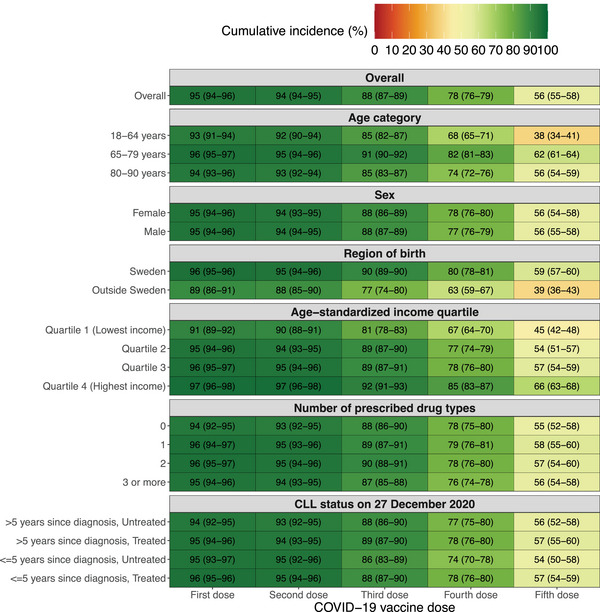
Cumulative incidences of uptake of first to fifth dose at the end of follow‐up by each independent variable. CLL = Chronic lymphocytic leukaemia, COVID‐19 = Coronavirus disease 2019. **Note**: Mortality and moving out of Sweden were considered competing events for each specific vaccine dose, that is, mortality before or after having received a first dose but not having received a second dose was a competing event for the analysis of the second dose, but not for the first dose. The numbers in each cell represent cumulative incidence in per cent (95% confidence interval) at the end of follow‐up.

Next, the cumulative incidences of uptake of the first to fifth doses at the end of follow‐up by age group, sex, region of birth, income quartile, number of prescribed drug types, and CLL status were estimated in individuals diagnosed with CLL before 27 December 2020 (Figure 3). The vaccine uptake was generally high for the first and second doses, whereas larger differences across age groups, regions of birth, and income quartiles were observed for the third to fifth doses. The cumulative incidence (95% CI) of uptake of the fifth dose was 39% (36%–43%) for individuals born outside Sweden compared with 59% (57%–60%) for individuals born in Sweden. The uptake was 45% (42%–48%) for individuals in income quartile 1 compared with 66% (63%–68%) for individuals in income quartile 4. Cumulative incidence curves, along with the corresponding *p*‐values obtained from Gray's test, are also presented for each variable in Figure S1.

**FIGURE 4:**
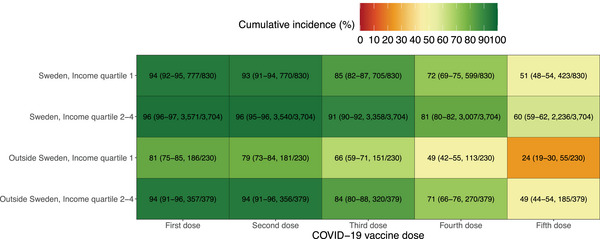
Cumulative incidences of uptake of first to fifth dose at the end of follow‐up in the four intersectional strata combining region of birth and income quartile. COVID‐19 = Coronavirus disease 2019. **Note**: Mortality and moving out of Sweden were considered competing events for each specific vaccine dose, that is, mortality before or after having received a first dose but not having received a second dose was a competing event for the analysis of the second dose, but not for the first dose. The numbers in each cell represent cumulative incidence in per cent (95% confidence interval, number vaccinated/total number of individuals) at the end of follow‐up.

The cumulative incidences of uptake of the first to fifth doses in the four intersectional strata combining region of birth, and income quartile are presented in Figure 4. Large differences in vaccine uptake were observed between the different strata, which tended to be more pronounced for the fourth and fifth vaccine doses. Differences between the lowest income quartile and the higher income quartiles were more pronounced among individuals born outside of Sweden compared with individuals born in Sweden. The cumulative incidence (95% CI) of uptake of the first dose was 81% (75%–85%) for individuals born abroad in income quartile 1 compared with 94% (91%–96%) for individuals born abroad in income quartiles 2–4. The corresponding numbers were 94% (92%–95%) and 96% (96%–97%) for individuals born in Sweden. For the fifth dose, the difference between the lowest income quartile and the higher income quartiles was 25% (24% vs. 49%) for individuals born abroad and 9% (51% versus 60%) for individuals born in Sweden. The *p‐*values obtained from Gray's test were significant for all five doses (Figure S2). Similar differences were also observed in the analyses of the 12 intersectional strata including age group, although less pronounced differences among individuals 80–90 years (Figures S3 and S4).

### 3.5 COVID‐19 vaccine uptake in individuals diagnosed with CLL from 27 December 2020

The vaccine uptake at the end of follow‐up was similar for individuals diagnosed with CLL during the study period (unvaccinated 3%, one dose 1%, two doses 5%, three doses 13%, four doses 25% and five doses 53%) compared with individuals diagnosed with CLL before the study period (unvaccinated 5%, one dose 1%, two doses 6%, three doses 11%, four doses 21% and five doses 56%).

## 4 DISCUSSION

In this nationwide register‐based cohort study including 6304 individuals with CLL in Sweden, we showed an overall high vaccine uptake for the first and second doses, with gradually decreasing uptake for the third to fifth doses. Even though the overall vaccine uptake is high in Sweden, people with CLL, as expected, had a significantly higher overall vaccine coverage compared to the age‐standardized population, plausibly since they were officially considered at risk for severe COVID‐19. Major sociodemographic disparities were however observed in vaccine uptake, with substantially lower uptake among younger individuals, individuals born abroad, and individuals in the lowest income quartile. These differences were especially pronounced in intersectional analyses, combining these three factors, and tended to be augmented for the booster doses compared with the primary vaccination series. No clear differences in vaccine uptake were observed between the individuals diagnosed with CLL before as compared to during the study period.

The vaccine uptake we observed for individuals with CLL was higher than the uptake reported in a British study of people with hematologic malignancies, although noted that people with CLL had the highest vaccination coverage of all groups of hematologic malignancies, with 41% and 51% coverage for the third and fourth dose, respectively [16]. However, in the British as well as our Swedish population, vaccine uptake was waning from dose 3 and onwards, with augmented sociodemographic disparities on vaccine uptake, as previously shown also in other sections of the Swedish population [12, 13, 14]. Deficient vaccination against COVID‐19 is especially worrisome in immunocompromised individuals such as those with CLL since multiple studies during the pre‐vaccination era of the pandemic have shown remarkably poor outcomes for individuals with CLL if infected with SARS‐CoV‐2, with hospitalization rates at 75%–90%, intensive care unit (ICU) admissions at 23–35% and mortality rates up to 38% [3, 4, 6]. And even if the situation has improved, due to increased coverage of COVID‐19 vaccinations, pre‐emptive treatment with monoclonal antibodies, early antiviral treatment etc, hospitalization rates at around 25%–50% have been reported and mortality rates have still been as high as 23% in some CLL populations during the Omicron era [33, 34].

Reasons for declining COVID‐19 vaccination after three doses might include concerns about vaccine safety and efficacy and/or lack of clear medical advice. Early results on vaccination against COVID‐19 have indeed been somewhat discouraging for people with CLL. Even though no more severe side effects have been observed in these individuals compared to the general population, only around 60% seroconversion rates and low antibody levels as well as impaired T cell activation were seen after 2 doses of mRNA vaccine [8, 9, 35, 36]. However, it has later been shown that with repeated booster vaccination, seroconversion rates and antibody levels increase, even though they remain lower than in healthy individuals, particularly if receiving BTK inhibitors [2, 7, 35]. Another reason for non‐vaccination may be a lack of motivation or the idea that “three doses should be enough”. However, an Israeli cohort study on patients with hematologic malignancies has shown that patients receiving mRNA vaccination 7–90 days prior to COVID‐19 were significantly less likely to develop severe COVID‐19, while no significant difference was seen for vaccination 7–180 days as compared to >180 days before infection, suggesting waning protection from vaccination 3–6 months after the last dose [37]. Furthermore, it has been observed in previous studies that a history of COVID‐19 is also associated with vaccination hesitancy. However, it has been demonstrated that even previously infected people benefit from booster vaccination [38, 39]. Repeated vaccination against COVID‐19 should thus be actively encouraged for individuals with CLL, and assuring that information of findings such as described above reaches patients – at medical appointments, via patient alliance channels or through targeted vaccination campaigns and vaccination policies – may help motivate patients to keep up with the vaccinations.

The fact that vaccine uptake is particularly low in individuals born outside of Sweden and having a lower disposable income, as seen also for example in Denmark, England, and the United States in people with hematologic malignancies as well as the general population [10–11, 15–16], suggest that vaccination programs are not adequately serving all parts of the community. Reasons for vaccine hesitancy in this part of the population may include a lack of trust in government and/or medical authorities, as well as a lack of trusted and/or accessible information. Linguistic and cultural barriers should be managed by providing targeted, adapted, and multilingual campaigns and specific outreach strategies. However, it should be noted that even if a relatively large proportion of individuals in our study born outside of Sweden and with a low income were not fully vaccinated, these individuals were quite few in total, with 88% of the entire cohort being born in Sweden and only 21% within the lowest income quartile. The number of individuals born outside of Sweden and in the lowest income quartile was only 230 in total. In fact, of those 253 individuals who were unvaccinated at the end of follow‐up (and diagnosed with CLL before the study started) 74% were born in Sweden. However, the highest proportion of unvaccinated individuals (38%) were found in the lowest income quartile, compared to 16% in the highest income quartile. Fifty‐three per cent of unvaccinated individuals were diagnosed with CLL > 5 years ago and 47% ≤ 5 years ago, 33% were considered untreated during the study period and 67% were treated, consistent with the baseline characteristics for the entire cohort. Sixty per cent of unvaccinated individuals were of the male sex, also consistent with the sex distribution for the entire cohort. This might suggest that more explicit medical advice is warranted for the entire CLL population.

A major strength of this study included the linkage of several national registers with high coverage, enabling comparisons of vaccine uptake during more than two years for individuals with CLL across different age groups and sociodemographic strata. Nonetheless, the study had some limitations. The study period for the fifth dose was rather short and uptake may thus be lower compared with a more up‐to‐date vaccine uptake in the population. Secondly, data were missing on around 250 individuals born before 1930 (>90 years of age), thus precluding generalizability for this age group. Furthermore, we did not have data on COVID‐19 vaccinations received outside of Sweden, which could lead to minor underestimations of vaccine uptake, in particular for individuals born abroad and getting vaccinated abroad. Finally, the herein observed results might not be directly generalizable to other settings, such as in lower‐ and middle‐income countries as well as countries with different COVID‐19 vaccination campaign strategies.

## 5 CONCLUSIONS

Our study demonstrates that even in a high‐risk profile population such as individuals with CLL, there are significant disparities in COVID‐19 vaccine uptake, where people with lower income and those born outside of Sweden are less likely to be fully vaccinated, and thus more vulnerable to severe COVID‐19. This is especially worrisome since people with CLL tend to need multiple booster doses to mount and sustain immune response from vaccination. These findings advise targeted information campaigns for these vulnerable patients in the ongoing vaccination program, emphasizing recent findings on the importance and efficacy of repeated booster vaccination in people with CLL as well as updated information on the COVID‐19 situation and corresponding risks. In addition, it should be ascertained that this information is easily accessible regardless of native tongue and educational level.

## AUTHOR CONTRIBUTIONS

All authors were involved in conceptualization, data collection, investigation, methodology, and resources. Funding was acquired by Lotta Hansson and Soo Aleman. Peter Bergman and Christina Carlander were responsible for the project administration. Lotta Hansson and Fredrik Granath supervised the project. Pontus Hedberg curated and visualized the data and wrote the original draft. Pontus Hedberg had full access to all the data in the study, performed the formal analyses, and validated the results. All authors contributed to reviewing and editing the manuscript and have read and approved the manuscript.

## CONFLICT OF INTEREST STATEMENT

Fredrik Kahn: Funding grants: CSL Behring. Peter Bergman: Speaking and lecture fees: CSL Behring, Takeda. Christina Carlander: Funding grants: Gilead Sciences Inc.; Consulting or advisory: Gilead Sciences Inc., ViiV Healthcare; Speaking and lecture fees: Gilead Sciences Inc., ViiV Healthcare. Soo Aleman: Funding grants: AbbVie, Gilead Sciences Inc.; Speaking and lecture fees: AbbVie, Biogen Inc., Gilead Sciences Inc., MSD. Lotta Hansson: Funding grants: IQVIA.

## ETHICS STATEMENT

The study was approved by the Swedish Ethical Review Authority (IDs 2022‐01793‐01 and 2023‐05877‐02).

## PATIENT CONSENT STATEMENT

The need for consent was waived by the Swedish Ethical Review Authority since analyses are based on retrospectively collected data from the administrative health registry.

## CLINICAL TRIAL REGISTRATION

The authors have confirmed clinical trial registration is not needed for this submission.

## NOVELTY STATEMENT

To the best of our knowledge, this is the first study addressing individual‐level sociodemographic disparities in coronavirus disease 2019 (COVID‐19) vaccine uptake among individuals with chronic lymphocytic leukaemia (CLL). We show that vaccine uptake even in this frail population is waning from the 3rd dose and onwards, and identify subpopulations less likely to pursue vaccination, such as low‐income earners and immigrants. Since individuals with CLL have been at serious risk of developing severe COVID‐19 during the pandemic, and have shown to often need several booster doses of vaccine to mount and sustain antibody responses due to disease‐ and treatment‐related immune defects, it is crucial to identify these individuals and to address them in future vaccination campaigns.

## Supporting information



Supporting Information

## Data Availability

All code used in the study is available upon request by the corresponding author. The individual participant data underlying this article were subject to ethical approval and cannot be shared publicly. Data from the deidentified administrative health registries are not freely available due to the protection of the personal integrity of the participants.

## Members of the CLHIP study group (last name alphabetical order):

Soo Aleman^1,2^, Peter Bergman^2,3,4^, Lisa Blixt^5,6^, Christina Carlander^1,2,7^, Sandra Eketorp Sylvan^8^, Mats Fredrikson^9^, Lotta Hansson^5,6^, Pontus Hedberg^1^, Fredrik Kahn^10^, Isabela Killander Möller^1^, Hannes Lindahl^4,11^, Åsa Nilsdotter‐Augustinsson^12^, Sofia Nyström^13^

## Affiliations

^1^Department of Medicine Huddinge, Karolinska Institutet, Stockholm, Sweden

^2^Department of Infectious Diseases, Karolinska University Hospital, Stockholm, Sweden

^3^Department of Laboratory Medicine, Karolinska Institutet, Stockholm, Sweden

^4^Department of Clinical Immunology and Transfusion Medicine, Karolinska University Hospital, Stockholm, Sweden

^5^Department of Oncology‐Pathology, Karolinska Institutet, Stockholm, Sweden

^6^Department of Haematology, Karolinska University Hospital, Stockholm, Sweden

^7^Department of Medical Epidemiology and Biostatistics, Karolinska Institutet, Stockholm, Sweden

^8^Department of Oncology‐Pathology, Karolinska Institutet, Stockholm, Sweden

^9^Department of Biomedical and Clinical Sciences and Forum Östergötland, Faculty of Medicine, Linköping University

^10^Department of Clinical Sciences, Division of Infection Medicine, Skåne University Hospital, Lund University, Lund, Sweden

^11^Department of Clinical Neuroscience, Karolinska Institutet, Stockholm, Sweden

^12^Division of Inflammation and Infection, Department of Biomedical and Clinical Sciences, Linköping University, Sweden

^13^Department of Clinical Immunology and Transfusion Medicine, and Department of Biomedical and Clinical Sciences, Linköping University Sweden
