# Supplementary material for: Uptake of the first to fifth doses of coronavirus disease 2019 vaccine in individuals with chronic lymphocytic leukaemia: A nationwide cohort study in Sweden
Source: EJHaem. 2025 Jan 6;6(1):e1077. doi: 10.1002/jha2.1077 (PMC11756972; doi:10.1002/jha2.1077)
Supplement: Supplementary file 1 — Supporting Information [file JHA2-6-e1077-s001.docx]

Supplementary material for

**Uptake of the First to Fifth Doses of COVID-19 Vaccine in Individuals with Chronic Lymphocytic Leukaemia: A Nationwide Cohort Study in Sweden**

**Authors:** Pontus Hedberg, Lisa Blixt, Fredrik Granath, Peter Bergman, Christina Carlander, Soo Aleman, Lotta Hansson on behalf of the CLHIP study group

Correspondence to lisa.blixt@regionstockholm.se

**Table of contents**

| **Content** | **Page** |
| --- | --- |
| Table S1. Definitions of study variables | 3-7 |
| Figure S1. Cumulative incidence curves of uptake of the first to fifth doses by each independent variable in individuals diagnosed with CLL before 27 December 2020 | 8-9 |
| Figure S2. Cumulative incidence curves of uptake of the first to fifth doses in the four intersectional strata combing region of birth and income quartile in individuals diagnosed with CLL before 27 December 2020 | 10 |
| Figure S3. Cumulative incidences of uptake of first to fifth dose at the end of follow-up in the 12 intersectional strata combing age group, region of birth, and income quartile in individuals diagnosed with CLL before 27 December 2020 | 11 |
| Figure S4. Cumulative incidence curves of uptake of the first to fifth doses in the 12 intersectional strata combing age group, region of birth and income quartile in individuals diagnosed with CLL before 27 December 2020 | 12 |

**Table S1. Definitions of study variables**

| **Variable** | **Data sources** | **Missing data** | **Definition** | **Time period** | **Possible values** |
| --- | --- | --- | --- | --- | --- |
| **Study outcomes** | | | | | |
| Receipt of first dose | NVR | No | A first vaccine dose given any time from 27 December 2020 to 28 February 2023 | 27 December 2020 to 28 February 2023 | Yes, No |
| Receipt of second dose | NVR | No | A second vaccine dose given any time from 15 January 2021 to 28 February 2023. 19 days since first dose if the first vaccine was BNT162b2 and NVX-CoV2372. 25 days since first dose if the first vaccine was mRNA-1273, AZD1222, and Ad26.COV2.S. | 15 January 2021 to 28 February 2023 | Yes, No |
| Receipt of third dose | NVR | No | A third vaccine dose given any time from 1 September 2021 to 28 February 2023. At least 56 days should have passed since the second dose. | 1 September 2021 to 28 February 2023 | Yes, No |
| Receipt of fourth dose | NVR | No | A fourth vaccine dose given any time from 21 January 2022 to 28 February 2023. At least 56 days should have passed since the third dose. | 21 January 2022 to 28 February 2023 | Yes, No |
| Receipt of fifth dose | NVR | No | A fifth vaccine dose given any time from 15 August 2022 to 28 February 2023. At least 56 days should have passed since the fourth dose. | 15 August 2022 to 28 February 2023 | Yes, No |
| **Competing events** |  |  |  |  |  |
| Death before vaccination | NCDR | No | Death before vaccine dose 1, 2, 3, 4, or 5 | 27 December 2020 to 28 February 2023 | Yes, No |
| Emigration before vaccination | TPR | No | Moving out of Sweden before vaccine dose 1, 2, 3, 4, or 5 | 27 December 2020 to 28 February 2023 | Yes, No |
| **Independent variables** | | | | | |
| Age | NCR | No | Age at start of study | 27 December 2020 | 28 to 90 years |
| Age category | NCR | No | Age category at start of study | 27 December 2020 | 18 to 64 years, 65 to 79 years, 80-90 years |
| Sex | NCR | No | Sex of individual | Birth | Male, Female |
| Region of birth | TPR | Yes for 1 individual | Region of birth | Birth | Sweden, Outside of Sweden, Missing |
| Age-standardized income quartile | LISA | Yes, for 1 individual | The income quartiles for each birthyear in the entire Swedish population was first calculated, and the study participants were then classified into income quartiles based on their income in relation to these population-based quartiles. The income variable was the total sum of incomes, including earned income, age-related pensions, compensation from unemployment insurance, sickness allowance etc. | 2020 | Quartile 1, Quartile 2, Quartile 3, Quartile 4, Missing |
| Number of prescribed drug types | SPDR | No | The number of the following drug types (ATC codes) prescribed from 27 December 2019 to 26 December 2020: Antidiabetics: A10.X Antihypertensives: C02.X, C03.X, C07.X, C08.X, C09.X Antithrombotics: B01.X Heart disease drugs:C01.X Immunosuppressive drugs: L01.X or L04.X Lipid-modifiers: C10.X Obstructive airway disease drugs: R03.X | 27 December 2019 to 26 December 2020 | 0, 1, 2, 3 or more |
| CLL treatment status | NCR, SPDR | No | The following drug classes were considered: Anti-CD20 antibodies BTK inhibitors BCL-2 inhibitors Chemotherapy Corticosteroids PI3K inhibitors.  For BTK inhibitors, BCL-2 inhibitors, chemotherapy, and PI3K inhibitors, all treatments given before 27 December 2020 (start of study) were considered.  For anti-CD20 antibodies, treatments given from 27 December 2019 to 27 December 2020 (last year before start of study) were considered.  For corticosteroids, treatments given from 28 September 2020 to 27 December 2020 (last 3 months) were considered. Corticosteroid injections and prescriptions with a daily dose corresponding to less than 10 mg of prednisone were not considered. This was <1.2 mg for Betamethasone, <1.5 mg for Dexamethasone, <8 mg for Methylprednisolone, <10 mg for Prednisolone, <40 mg for Hydrocortisone, and <50 mg for Cortisone acetate.  Individuals who had not received any of these treatments were classified as untreated. |  | Treated, Untreated |

**Abbreviations:** ATC=Anatomical Therapeutic Chemical, BCL-2=B-cell lymphoma 2, BTK=Bruton Tyrosine Kinase, CD=Cluster of differentiate, LISA=Longitudinal Integrated Database for Health Insurance and Labour Market Studies, NCDR=National Cause of Death Register, NCR=National CLL Registry NVR=National Vaccination Register, PI3K=Phosphoinositide 3-kinase, SPDR=National Prescribed Drug Register, TPR=Total Population Register

**Figure S1. Cumulative incidence curves of uptake of the first to fifth doses by each independent variable in individuals diagnosed with CLL before 27 December 2020**

**
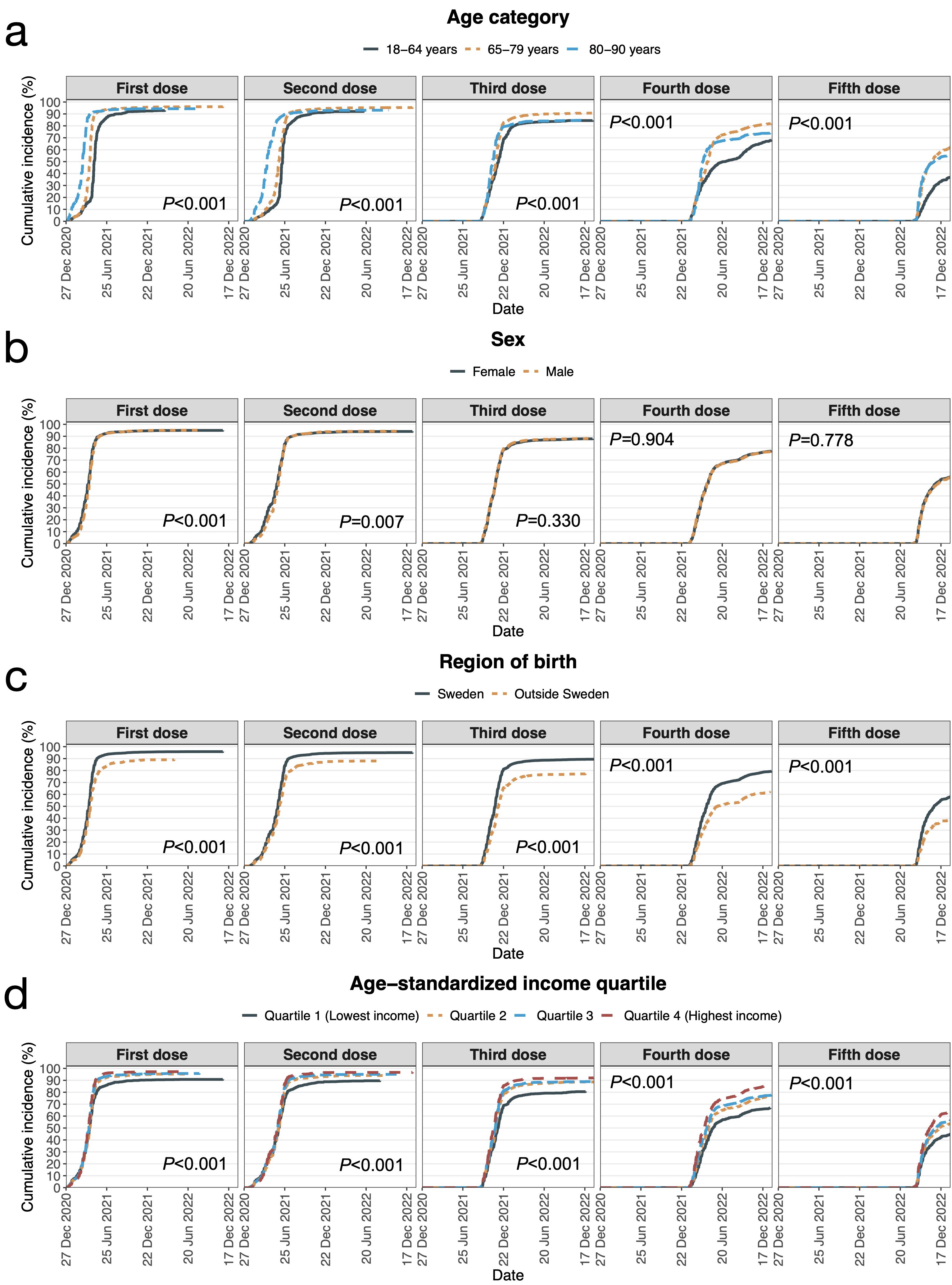
**

**
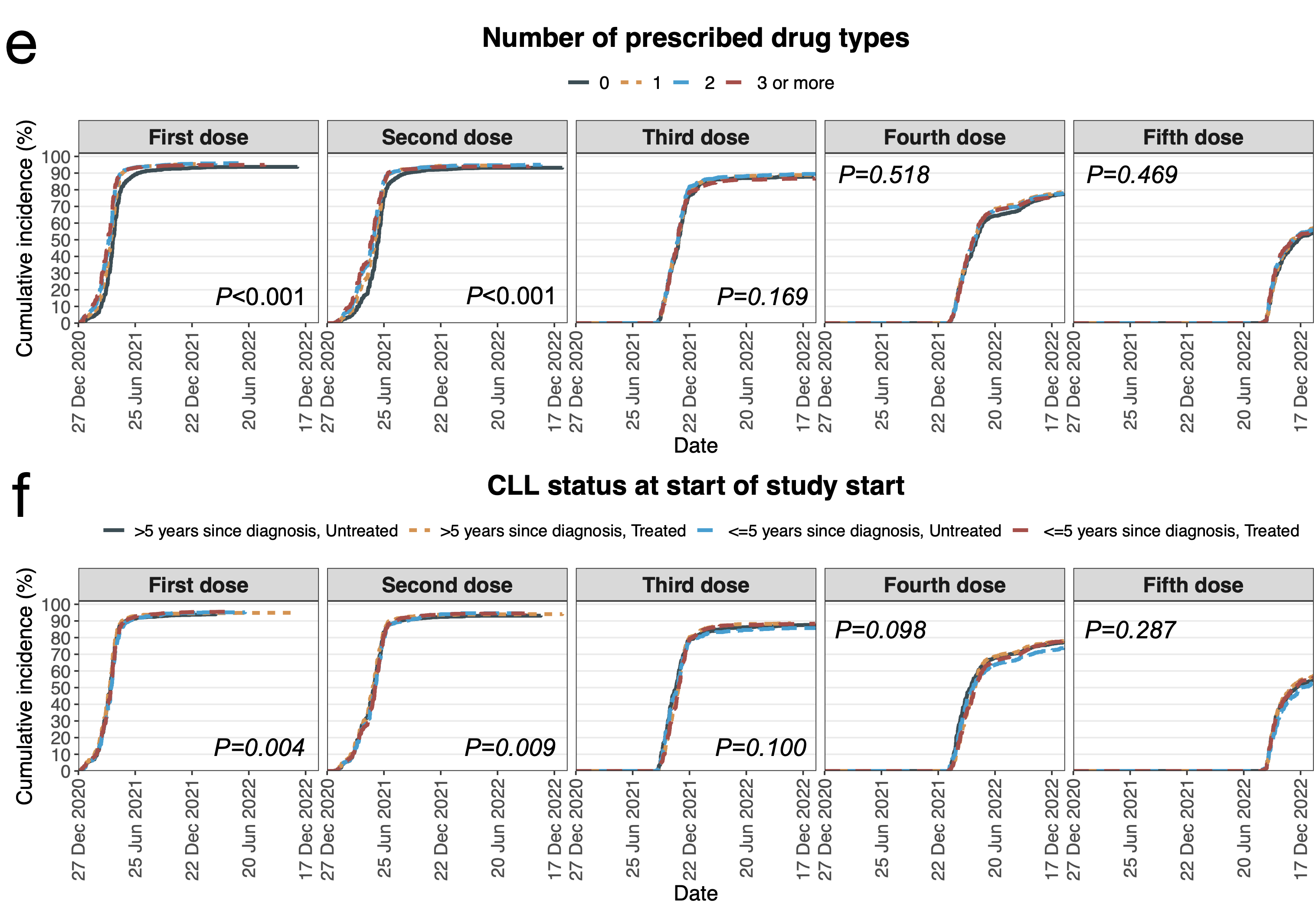
**

**Abbreviations:** CLL=Chronic lymphocytic leukaemia, COVID-19=Coronavirus disease 2019

**Note:** Mortality and moving out of Sweden were considered competing events for each specific vaccine dose, i.e., mortality before after having received a first dose but not having received a second dose was a competing event for the analysis of the second dose, but not for the first dose. The *P* value was obtained from Gray’s test for each vaccine dose.

**Figure S2. Cumulative incidence curves of uptake of the first to fifth doses in the four intersectional strata combing region of birth and income quartile in individuals diagnosed with CLL before 27 December 2020**

**
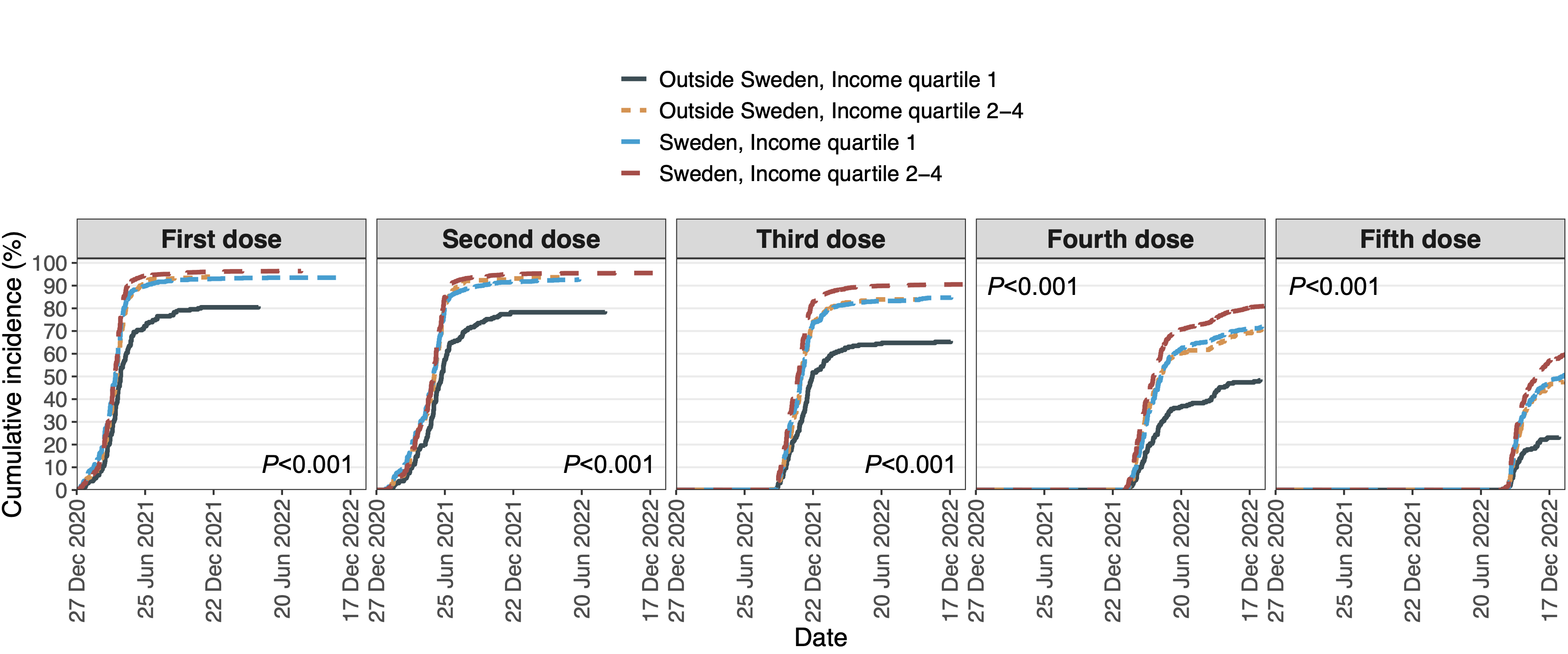
**

**Abbreviations:** COVID-19=Coronavirus disease 2019

**Note:** Mortality and moving out of Sweden were considered competing events for each specific vaccine dose, i.e., mortality before after having received a first dose but not having received a second dose was a competing event for the analysis of the second dose, but not for the first dose. The *P* value was obtained from Gray’s test for each vaccine dose.

**Figure S3. Cumulative incidences of uptake of first to fifth dose at the end of follow-up in the 12 intersectional strata combing age group, region of birth, and income quartile in individuals diagnosed with CLL before 27 December 2020**

**Abbreviations:** COVID-19=Coronavirus disease 2019

**Note:** Mortality and moving out of Sweden were considered competing events for each specific vaccine dose, i.e., mortality before after having received a first dose but not having received a second dose was a competing event for the analysis of the second dose, but not for the first dose. The numbers in each cell represent cumulative incidence in percent (95% confidence interval, number vaccinated/total number of individuals) at the end of follow-up.

**Figure S4. Cumulative incidence curves of uptake of the first to fifth doses in the 12 intersectional strata combing age group, region of birth and income quartile in individuals diagnosed with CLL before 27 December 2020**

**
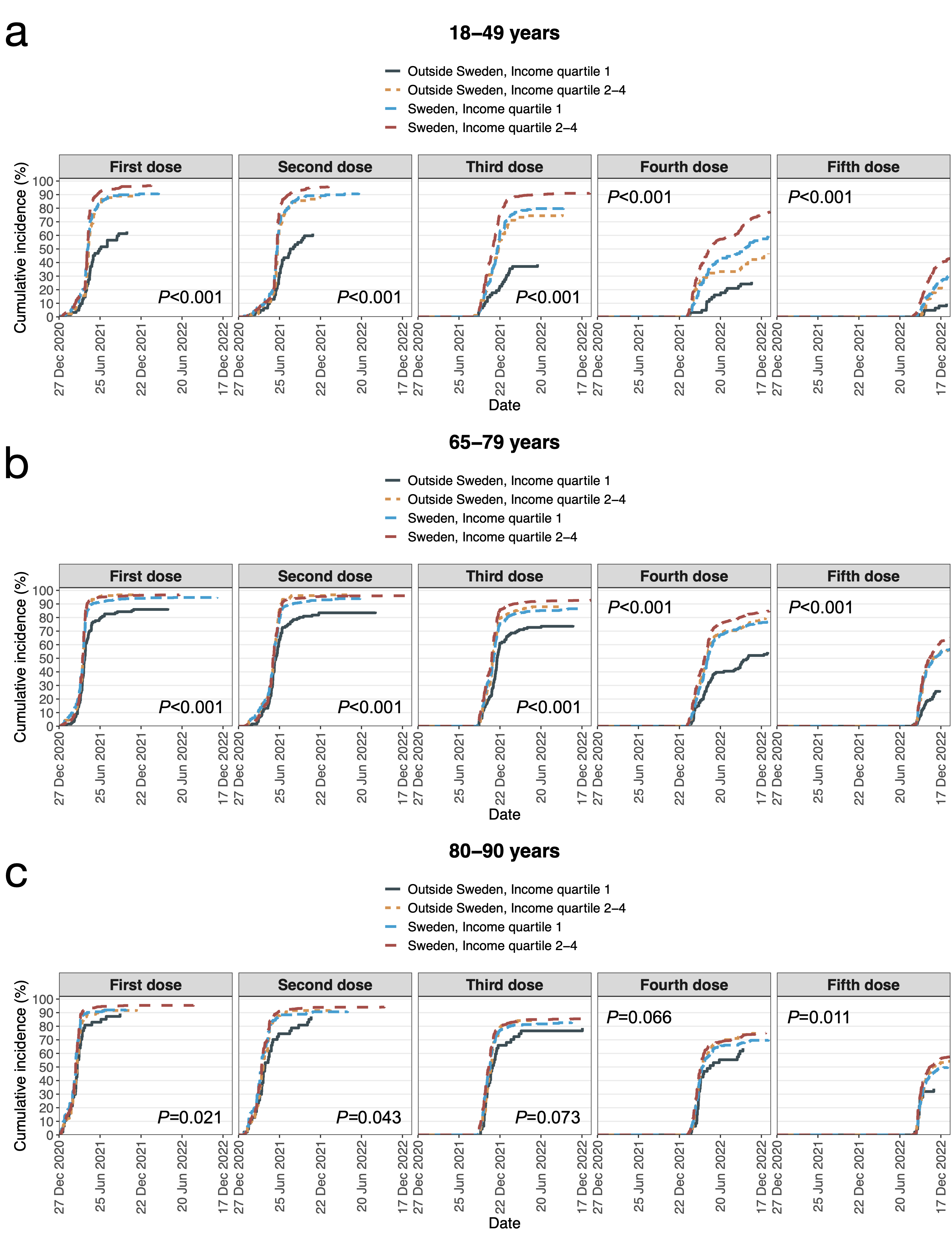
**

**Abbreviations:** COVID-19=Coronavirus disease 2019

**Note:** Mortality and moving out of Sweden were considered competing events for each specific vaccine dose, i.e., mortality before after having received a first dose but not having received a second dose was a competing event for the analysis of the second dose, but not for the first dose. The *P* value was obtained from Gray’s test for each vaccine dose.
